# Contribution of Genetic Factors to Sjögren's Syndrome and Sjögren's Syndrome Related Lymphomagenesis

**DOI:** 10.1155/2015/754825

**Published:** 2015-10-15

**Authors:** Adrianos Nezos, Clio P. Mavragani

**Affiliations:** Department of Physiology, Medical School, University of Athens, Mikras Asias 75, 11527 Athens, Greece

## Abstract

We aimed to summarize the current evidence related to the contributory role of genetic factors in the pathogenesis of Sjögren's syndrome (SS) and SS-related lymphoma. Genes within the major histocompatibility complex (MHC) locus previously considered conferring increased susceptibility to SS development have been also revealed as important contributors in recent genome wide association studies. Moreover, genetic variations outside the MHC locus involving genes in type I interferon pathway, NF-*κ*B signaling, B- and T-cell function and methylation processes have been shown to be associated with both SS and SS-related lymphoma development. Appreciating the functional implications of SS-related genetic variants could provide further insights into our understanding of SS heterogeneity, allowing the design of tailored therapeutic interventions.

## 1. Introduction

Sjögren's syndrome (SS) is the second most common systemic autoimmune disease (after rheumatoid arthritis, RA), with a prevalence of about 0.5% in the general population. It occurs primarily in perimenopausal women (at a ratio of women to men of 9 : 1) [1, 2]. Key features of the disease include infiltration of exocrine glands (predominantly salivary and lacrimal) by lymphocytes, production of inflammatory cytokines, and the activation of B lymphocytes and the production of autoantibodies. At the level of exocrine glands, loss of secretory activity has been observed leading to the characteristic symptoms of dry mouth and dry eyes. Other systems or organs such as musculoskeletal system (arthralgias, myalgias, and nonerosive arthritis of small joints), lungs, liver, skin, and kidneys can also be involved [3]. Compared to the general population, patients with SS exhibit significantly increased risk of developing B-cell lymphoproliferative disorders (usually B-cell lymphoma involving the mucosa-associated lymphoid tissue (MALT)), which affects about 5–10% of patients leading to significant increased morbidity and mortality rates of these patients [4]. Several clinical and laboratory manifestations have so far been proposed as adverse predictors for malignant disease and include features related to deposition of immune complexes (palpable purpura, peripheral neuropathy, low levels of complement C4, and cryoglobulinemia), swelling of the parotid glands, as well as high lymphocytic scores, and the presence of ectopic germinal centers in minor salivary gland biopsies [5]. The causative mechanisms of SS have not been fully elucidated. However, based on the current pathogenetic model, the interaction of both genetic, epigenetic [6, 7] and environmental factors seems to contribute to disease development [8]. Viruses, especially the Epstein-Barr virus (EBV) [9] and Coxsackievirus [10], hormones (low estrogen levels seen in perimenopausal women), stress [11], and occupational exposures [12] have been all considered as the main environmental triggers for disease onset. In the current review article, we will mainly focus on the contributory role of genetic influences in the development of the SS as well as in SS-related lymphoproliferation, a major disease complication.

## 2. Genetic Factors Associated with Sjögren's Syndrome

Familial clustering and coaggregation with other autoimmune disorders in SS has been long considered [13–16]. In an Italian multicenter case control study [17], the risk of SS development among first-degree relatives with autoimmune disease was sevenfold higher compared to controls. Of note, these first-degree relatives of SS patients had a higher risk of autoimmune disease compared to subjects without first-degree relative affected by SS. A large recent study in Taiwanese population confirmed previous observations showing that first-degree relatives of SS patients had an increased risk of SS development as well as of other autoimmune disorders, mainly systemic lupus erythematosus (SLE), RA, systemic sclerosis, and type 1 diabetes (T1D), compared to the general population [14]. Of interest, siblings of affected individuals demonstrated the highest relative risk for SS development compared to other first-degree relatives (parents and offsprings), implying both genetic influences and shared environmental exposures as contributory factors to disease development [14].

Since the 1970s, strong associations between specific alleles of the major histocompatibility complex (MHC) and SS development have been suggested [18, 19]. Over the last decade, high throughput technologies allowed the confirmation of the dominant role of MHC alleles in the pathogenesis of the disease with novel genetic variants outside the MHC locus emerging as susceptibility factors [20]. The latter seem to be involved in signaling pathways of natural and acquired immunity, inflammatory responses and cellular apoptosis.

### 2.1. The Role of MHC Complex

Though initially recognized as major determinants of tissue rejection, MHC genes have been soon after appreciated as critical contributors to the pathogenesis of autoimmune disorders as well. They encode components of the human leukocyte antigen (HLA) system [21] including HLA class I (A, B, and C) and class II (DR, DQ, and DP), which present endogenous and exogenous antigens to T lymphocytes, respectively. MHC class II molecules, especially those which encode HLA-DR and HLA-DQ antigens, have been proposed as the most important genes associated with SS susceptibility. The first genetic study on SS revealed an association between SS and HLA-DR3 (which was in linkage disequilibrium (LD) with the class I allele HLA-B8) in Caucasian SS patients [22]. Subsequent reports highlighted the association between SS and the HLA-D locus [18, 23], with a diverse distribution between primary SS (high frequency of the alleles HLA-DRw3 and HLA-B8) and secondary SS (increased frequency of allele HLA-DRw4) [24], as well as in patients with SS characterized by the presence of Raynaud's phenomenon (increased frequency of alleles HLA-DRw3 and HLA-DRw4) [25]. Subsequent studies in other ethnic populations confirmed HLA associations with SS susceptibility [26–41], with DRB1^*∗*^04:05-DQB1^*∗*^04:01 being a risk allele in Japanese populations [36], DRB1^*∗*^08:03-DQB1^*∗*^06:01 in Chinese populations [36], DR3 and DR11 in Spanish populations [42], and DRB1^*∗*^11:01, DRB1^*∗*^11:04, DQB1^*∗*^03:01, and DQA1^*∗*^05:01 in Israeli Jews and Greeks [43, 44] (Summarized in Table 1). In a subsequent meta-analysis [21], HLA class II alleles DRB1^*∗*^03:01, DQA1^*∗*^05:01, and QB1^*∗*^02:01 were shown to predispose to disease development, while DQB1^*∗*^05:01 exhibited a protective role [21]. Two recent large genome wide association studies (GWAS), in Caucasian [45] and Han-Chinese population [46], confirmed the strong influence of HLA locus on SS. Further studies are required on the functional role of the HLA polymorphic regions in SS pathogenesis as well as their possible associations with disease diagnosis and/or prognosis.

A functional deletion of 6.7 Kb in the gene leukocyte immunoglobulin-like receptor subfamily A member 3 (LILRA3) has been also associated with autoimmune diseases. LILRA3 is a soluble receptor of class I MHC antigens involved in the regulation of immune function. It has been found in SS patients of both Caucasian [47] and Chinese [48] origin, as well as in other autoimmune diseases including multiple sclerosis [49], RA [50], and SLE [48].

### 2.2. Genetic Factors Associated with SS outside the MHC Locus

In the following years, research has been directed to the investigation of single nucleotide polymorphisms (SNPs) in genes outside the MHC locus (summarized in Table 2 [51–68]), already found to be associated with other autoimmune diseases such as SLE [69]. These novel SS-associated genetic variants (outside the MHC locus) can be roughly classified into three main groups depending on the implicated signaling pathway [20]. The first group consists of variants in genes involved in the activation of the interferon (IFN) signaling pathway. The second group includes important genes affecting B-cell function and autoantibody production. Specific autoantibodies have been found in approximately two-thirds of SS patients and genetic contribution has been proposed. HLA class II SS-related phenotype has been associated with the presence of autoantibodies in various studies [33, 35, 40]. SNPs in the 5′ untranslated region of the BAFF [61] gene as well as in GTF2I [70] and genes implicated in the NF-*κ*B pathway [45] have been also associated with the presence of autoantibodies. Finally, the third one contains apoptotic and inflammatory genes, which participate in the NF-*κ*B signaling pathway.

#### 2.2.1. Genes Associated with Interferon Pathways

Gene expression studies in SS patients over the last decade revealed upregulation of IFN-inducible genes (the so-called IFN signature) at the level of peripheral blood and affected salivary gland tissues in a substantial proportion of these individuals [71]. Moreover, recent data revealed that both type I (IFN*α*/*β*) and type II (IFN*γ*) IFN signatures are upregulated in both peripheral blood and minor salivary gland tissues derived from SS patients while the IFN*γ*/IFN*α* mRNA ratio in diagnostic salivary gland biopsies could predict the in situ lymphoma development in the setting of SS [72, 73]. While the mechanisms leading to this activation remain under investigation, several genetic variants in genes implicated in the IFN pathway have been designated as potential contributors [74].

*Interferon Regulatory Factor 5 (IRF5)*. IRF5 is a transcription factor involved in type I IFN induction following TLR ligation [75]. Several polymorphisms of the IRF5 gene have been previously shown to either increase or decrease SLE susceptibility [74, 76]. Initial studies on SS revealed the IRF5 polymorphism rs2004640 (creates an alternate splice site (exon 1B) in the first exon) as predisposing factor to disease development in both Scandinavian and French cohorts [51, 52]. Another strong signal of association was observed between the insertion/deletion (in/del) of the CGGGG sequence in the IRF5 gene promoter in both SS and other autoimmune diseases [52, 53, 77]. This CGGGG in/del is part of a polymorphic repetitive DNA region, which includes either 3 or 4 CGGGG repeats; the insertion of an additional CGGGG unit (the 4 × CGGGG allele) is the risk allele associated with increased IRF5 transcription in peripheral blood mononuclear cells (PBMCs) and cultured epithelial cells derived from the salivary glands of SS patients, possibly through the addition of Sp1 binding site in the gene promoter of the 4R allele [53, 77]. Moreover, reovirus infection of salivary gland epithelial cells from SS patients carrying the 4R allele further increased IRF5 expression at mRNA level [53]. Taken together, these findings suggest a possible association between the IRF5 gene variants and the induction of type I IFNs (through the induction of the IRF5 gene expression after a viral infection) that could lead to the robust activation of the immune system in salivary glands (target organ) as an early event in SS pathogenesis.

Recent studies revealed genetic association of Transportin-3 (TNPO3), an IRF5 neighboring gene that encodes a nuclear receptor involved in the import of splicing factors in the nucleus, with both SLE and SS susceptibility with specific variants spanning the IRF5-TNPO3 locus being identified [45, 76].

*Signal Transducer and Activator of Transcription 4 (STAT4)*. The transcription factor STAT4 is primarily involved in the signal transduction induced by the cytokines interleukin- (IL-) 12 and IL-23 leading to differentiation of T helper (Th) naïve cells towards a Th1 phenotype and subsequent production of IFN*γ* [78]. STAT4 intronic variants, namely, rs7582694 and rs7574865, have been associated with SS development in four candidate gene association studies [52, 55, 56, 79]. Subsequent GWAS studies in SS patients with both European and Chinese descent confirmed STAT4 locus as an important determinant of SS susceptibility [45, 46]. While rs7582694 risk variant has been associated with increased expression of several IFN-inducible genes in SS patients [79], PBMC derived from lupus patients harboring the risk variant of rs7574865 demonstrated increased sensitivity to IFN*α* effects [78]. 

*Interleukin 12A (IL12A)*. The recent GWAS in the Caucasian [45] but not Chinese population revealed an important association of IL12A gene polymorphisms with SS. The IL12A is a cytokine that forms a heterodimer with the IL12B subunit inducing through STAT4 the differentiation of naïve T-cells in T helper 1 cells which promotes immune response through IFN*γ* production by T helper 1 cells [80]. 

*Natural Cytotoxicity Triggering Receptor 3 (NCR3)*. NCR3/NKp30 is a natural killer (NK) specific receptor regulating the cross talk between NK and dendritic cells as well as type II IFN secretion [81]. The minor allele of the rs11575837 polymorphism within the promoter of NCR3 gene has been found as protective allele for SS development that is associated with reduced NCR3 gene transcription. Compared to controls, SS patients who lacked this polymorphism demonstrated higher circulating levels of the NCR3 ligand and demonstrated higher focus scores in salivary gland biopsy [57]. 

*Protein Tyrosine Phosphatase Nonreceptor 22 (PTPN22)*. PTPN22 gene encodes the protein lymphocyte tyrosine phosphatase (Lyp) previously shown to be implicated in both adaptive (inhibition of T-cell receptor (TCR) and B-cell receptor (BCR) signaling) and innate immune responses (type I interferon (IFN) production by myeloid cells through TLR ligation) [82]. A single nucleotide polymorphism (SNP) of the PTPN22 gene 1858C>T (rs2476601) leading to substitution of arginine (R) by tryptophan (W) at position 620 has been previously shown to increase susceptibility to several autoimmune diseases including T1D, SLE, and RA [83–85]. Although the underlying mechanisms leading to autoimmunity are not clearly delineated, a break in B- and T-cell tolerance through altered BCR and TCR signaling, enhancement of T helper follicular cells, and dampened type I IFN responses leading to a proinflammatory microenvironment have been all shown to contribute to autoimmune pathogenesis reviewed in [82].

In regard to SS, available data so far is rather conflicting. In contrast to a French report [58] in which no association with SS development was detected, studies in both Colombian [59] and Greek populations identified a strong association with SS susceptibility, particularly in patients characterized by low IFN signatures (manuscript in preparation). On this basis, we postulate that the apparent discrepancies between different studies are related to IFN status of SS patients included. This finding implies an additional shared etiological origin in autoimmune disorders, with a putative role of genetic contributors as determinants of distinct IFN patterns in patients with autoimmune diseases.

#### 2.2.2. Genes Involved in B-Cell Function

*B-Lymphocyte Kinase (Blk)*. The kinase Blk is a member of the family of the src tyrosine kinase, which seems to be involved in signaling and differentiation of B lymphocytes [86]. Common polymorphisms in the Blk and in the neighboring family with sequence similarity 167, member A (FAM167A) genes have been found to predispose to SLE [87], systemic sclerosis [88], RA [89], and recently SS [54, 60, 90]. Two main SNPs associated with SS development include rs12677843 (located in intron 1 of the Blk gene) and rs12549796 (second intron of FAM167A). These SNPs have been found in partial LD (*r* ^2^ = 0.29). The functional implication of these SNPs in SS remains unknown, although previous studies on SLE showed an association between the presence of risk alleles with decreased Blk mRNA levels and increased FAM167A mRNA levels in transformed B lymphocytes [87]. The association of Blk/FAM167A polymorphisms with SS was also suggested in a large GWAS study in Caucasian populations [45].

*B-Cell Activating Factor (BAFF)*. BAFF is an important cytokine that promotes survival and proliferation of B-cells. Previously published data support a role for several haplotypes in the 5′ regulatory region of BAFF gene in autoantibody positive SS and increased serum BAFF levels [61] as well as in distinct (both low and high risk for lymphoma development) SS phenotypes [62]. 

*Chemokine (C-X-C Motif) Receptor 5 (CXCR5)*. In a GWAS Caucasian study, chemokine receptor CXCR5 gene variants were found to confer protection against SS development [45]. The chemokine receptor CXCR5 detected in both circulating B-cells and activated CD4^+^ cells contributes to B- and T-cell migration in peripheral lymphoid as well as in inflamed peripheral organs, upon ligation with the CXCL13 chemokine [91, 92]. The latter has been previously found to be upregulated in salivary gland tissues derived from SS patients leading eventually to preferential retention of memory CXCR4^+^CXCR5^+^ B-cells in the SS derived salivary gland infiltrates [93].

*Early B-Cell Factor 1 (EBF1)*. In a large candidate gene association study in SS patients of Scandinavian origin [54], genetic variants of the* EBF1* gene (previously shown to be involved in antigen independent changes of B-cell differentiation [94]) have been found to confer increased risk for SS.

*Ox40 Ligand/Tumor Necrosis Factor Superfamily 4 (Ox40L/TNFSF4)*. Ox40L (or TNFSF4), a TNF family ligand member, expressed on activated dendritic cells, endothelial cells, and the B-cell surface, has been previously shown to get involved in B-cell activation through interaction with Ox40-positive T-cells [95, 96]. Genetic variants of Ox40L have been previously associated with susceptibility to SLE (in association with increased transcript and protein levels) and scleroderma, but not with primary biliary cirrhosis or SS after Bonferroni corrections in a Han-Chinese population [63, 97, 98]. However, in a Scandinavian study, two SNPs (namely, rs1234315 and rs1234314) located in the 5′-untranslated region of the gene Ox40L have been found to be significantly associated with SS [54]. 

*General Transcription Factor 2I (GTF2I)*. An interesting finding from a large study (GWAS) in Han-Chinese [46] but not in European population revealed that a polymorphism in the GTF2I gene (namely, rs117026326) (encodes a transcription factor involved in both T-cell signaling [99] and activation of immunoglobulin heavy-chain transcription upon B-lymphocyte activation [100]) is strongly associated with SS development with overall risk (OR) scores higher than other SS-associated identified genes including MHC-II genes, STAT4, and TNFAIP3 [46]. This finding was also confirmed in another study in Chinese population and was linked to the presence of anti-Ro/SSA autoantibodies [70].

#### 2.2.3. Genes Involved in the NF-*κ*B Pathway

*Tumor Necrosis Factor-Alpha Induced Protein 3 (TNFAIP3)*. TNFAIP3 gene encodes the A20 protein, an enzyme with ubiquitination activity that appears to play an important role in the regulation of inflammation through the NF-*κ*B pathway. A20 protein is expressed at low levels on most of the cells but is rapidly induced after activation of NF-*κ*B, acting as a negative feedback regulating both inflammation and apoptosis [101]. Experiments on mice revealed that A20 is important for survival and normal development since A20-deficient mice fail to regulate TNF induced NF-*κ*B activation and die early due to multiorgan inflammation and cachexia [102]. Several genetic variants of the TNFAIP3 gene have been associated with autoimmune diseases including SS [64]. The coding TNFAIP3 polymorphism, namely, rs2230926, which changes the amino acid sequence from phenylalanine (Phe) to cysteine (Cys) at position 127 has been previously found to confer increased risk for SLE [103]. Functional analysis showed that the rs2230926 minor allele, which predisposed to disease, is less effective in inhibiting the activity of NF-*κ*B after induction by TNF [103]. The association of TNFAIP3 rs2230926 polymorphism with SS has been recently confirmed by two large studies (GWAS) in both Caucasian [45] and Chinese population [46].

*TNFAIP3-Interacting Protein 1 (TNIP1)*. Of note, polymorphisms of the* TNIP1* gene, a molecule which interacts with the TNFAIP3 gene regulating the NF-*κ*B activation, have been recently found to confer increased risk to SS [54, 66] and other autoimmune diseases [104, 105]. The role of TNIP1 polymorphisms in SS development was also confirmed in a large GWAS study in Caucasian population [45]. 

*Lymphotoxin Gene A (LTA)*. Polymorphisms of the lymphotoxin gene A (LTA), located on locus LTA/LTB/TNF and related to the activation of the NF-*κ*B pathway as well as inflammation, have been found to increase the risk of SS [67].

*Chemokine (C-C Motif) Ligand 11 (CCL11)*. Finally, the CCL11 (eotaxin) is a chemokine with important role in SS. The expression of CCL11 has been found to be regulated by the NF-*κ*B pathway and specific polymorphisms in the CCL11 gene have been associated with ectopic germinal center-like structures present in salivary gland tissues of a proportion of SS patients who are found to be at risk of lymphoma development [106].

### 2.3. Animal Models for the Study of Genetic Predisposition to SS

Animal models are useful tools for elucidating the etiopathogenetic mechanisms of various autoimmune diseases including SS. Over the last decade, various murine models have been proposed in an attempt to explore the early initiating and subsequent events leading to disease development. Spontaneous or transgenic murine models which are prone to develop Sjögren's syndrome-like symptoms during lifetime include, among others (as reviewed recently in [107]), (NZB/NZW)F1, MRL, NOD, NOD-Aec1Aec2, Baff Tg, Opn Tg, and Act1^−/−^. The latter has been recently found to develop a disorder which closely resembles Sjögren's syndrome in association with lupus. The Act1-deficient mice are characterized by marginal zone-like B-lymphocyte accumulations, salivary and lachrymal gland inflammation, and production of anti-Ro/SSA and anti-La/SSB autoantibodies [108]. Act1 is a negative regulator of BAFF and CD40 molecules (both implicated in B-cell survival and activation) while recent findings proposed it to be a critical component of the IL-17 signalling pathway [109]. Of interest, several SNPs around the TRAF3IP2 gene (which encodes the Act1 protein) have been recently found to confer increased risk to lupus and may play an important role in the induction of the interferon pathway (interferon-*β*, interferon inducible genes), which is relevant in the context of autoimmune diseases, like lupus and SS [110]. Taken together, these findings indicate the putative role of the SNPs in the TRAF3IP2 gene in the development of histological and serological features of SS.

### 2.4. Genetic Factors Associated with Sjögren's Syndrome Related Lymphomagenesis

Lymphocytic infiltration of the exocrine glands and ectopic formation of germinal centers have been considered as the sine qua non of lymphoma development. B-cell hyperactivity, the hallmark of SS, molecular events affecting B-cell function and survival, and the deregulation of the NF-*κ*B pathway have been recently proposed as potential factors leading to lymphoma development [111]. Chronic antigenic stimulation of autoreactive B-cells and tumorigenic events such as chromosomal translocation and gene mutations/polymorphisms have been suggested as possible mechanisms underlying neoplastic diversion in the setting of SS. Regarding oncogenic mechanisms, the presence of the translocation *t*(14; 18) (leading to overexpression of Bcl-2, an antiapoptotic gene promoting B-cell survival) has been detected in 5 of 7 salivary gland biopsies of patients with Sjögren's syndrome who developed lymphoma and in none of the 50 corresponding biopsies of patients with the syndrome not associated with lymphoma [112]. Furthermore, mutations of tumor suppressor gene p53 are possibly associated with the occurrence of lymphoma in patients with SS [113].

Additionally, somatic mutations and polymorphisms in the TNFAIP3 gene have been also reported in several types of lymphomas [114] including lymphomas of mucosal marginal zone (MALT), which is the major type of SS-related lymphoproliferative disease. In a recent study, the rs2230926 TNFAIP3 polymorphism along with other genetic alterations has been found to be associated with SS-related lymphoproliferation, especially of MALT type, while functional assays found that this polymorphism is associated with increased activation of the NF-*κ*B pathway [65].

Another study failed to provide evidence for the presence of MyD88 L265P gene mutation (a nonsynonymous change at amino acid position 265 from leucine to proline (L265P)) in patients with SS with and without lymphoma [115]. MyD88 is an adaptor protein leading to NF-*κ*B activation through TLR, IL-1R, and IL-18 signaling, which has been previously shown to be implicated in patients with Waldenström's macroglobulinemia (WM) and other haematological malignancies [116, 117]. The absence of mutation in SS patients with or without lymphoma suggests that probably there are different pathogenetic mechanisms in lymphoproliferation in the setting of SS [115].

Given that deregulation of B-cell activation has been postulated as fundamental event in both autoimmunity and B-cell lymphomagenesis, the BAFF/BAFF-R axis attracted our research interest. Specific haplotypes of the BAFF gene could discriminate SS patients with lymphoma from SS patients without lymphoma and healthy controls [62] and a functional mutation His159Tyr of the BAFF receptor (BAFF-R), previously found to confer an increased risk in patients with NHL through activation of the alternative NF-*κ*B pathway [118], has been found to be more prevalent in SS population compared to healthy controls. Of interest, more than two-thirds of SS patients complicated by MALT type NHL with an age at SS diagnosis between 3rd and 4th decade carried this mutation [68].

The role of known polymorphisms of the methylenetetrahydrofolate reductase (MTHFR), gene, an enzyme necessary for the DNA synthesis and methylation, which have been previously associated with NHL development [119, 120] and autoimmune diseases [121], has been also investigated. MTHFR polymorphisms have been found to be associated with both SS and SS non-MALT NHL development in association with methylation alterations, implying genetic and epigenetic abnormalities as common pathogenetic pathways in both benign autoimmunity and malignant transformation (Fragioudaki et al., in preparation).

## 3. Discussion/Conclusions

While growing evidence over the last years supports a genetic contribution to SS susceptibility, the majority of genetic variants seem to have weak or moderate effect (except, perhaps, for HLA locus), implying an additional role for the environmental insults such as viruses, hormones, and stress in disease pathogenesis. Given that the vast majority of these genetic loci have been also detected as susceptibility factors in other autoimmune disorders, shared mechanisms leading to deregulation of the immune system imply a central role in autoimmune pathogenesis. Heterogeneity of SS clinical expression from local disease confined to exocrine glands to lymphoma development should be always taken into account when genetic studies are designed, since distinct operating immune pathways underlie distinct clinical phenotypes. Further multicenter efforts exploring genetic, epigenetic, and environmental interactions are warranted to further clarify the pathogenesis of the syndrome.

## Figures and Tables

**Table 1:** Genetic associations of the HLA alleles with Sjögren's syndrome susceptibility.

Study	Year	Population	Sample size (patients/controls)	Associated HLA alleles
Chused et al. [18]	1977	American Caucasian	110 (19/91)	HLA-Dw3
Fye et al. [22]	1978	American Caucasian	115 (19/96)	HLA-Dw3-HLA-B8
Moutsopoulos et al. [23]	1978	American Caucasian	208 (24/184)	B lymphocytes immune response associated (Ia) antigens
Moutsopoulos et al. [24]	1979	American Caucasian	206 (22/184)	HLA-DRw3-HLA-B8
Manthorpe et al. [27]	1981	Danish	32 (32/—)	HLA-Dw2
Mann and Moutsopoulos [25]	1983	American Caucasian	52 (25/27)	HLA-DRw3-HLA-B8
Molina et al. [28]	1986	American Caucasian	694 (68/626)	HLA-B8 HLA-DR3 DRw52
Moriuchi et al. [29]	1986	Japanese	135 (21/114)	DRw53
Vitali et al. [30]	1986	Italian	90 (28/62)	DR3
Papasteriades et al. [26]	1988	Greek	218 (46/172)	DR-5
Pease et al. [31]	1989	British Caucasian	141 (41/100)	DR-3 DRw52
Morling et al. [41]	1991	Danish	19 (19/—)	DQA1^*∗*^0501-DQB1^*∗*^0201-DQA1^*∗*^0301
Kang et al. [36]	1993	American Caucasian	210 (75/135)	DRB1^*∗*^03-DRB3^*∗*^0101-DQB1^*∗*^0201-DQA1^*∗*^0501
Kang et al. [36]	1993	Chinese	87 (45/42)	DRB1^*∗*^0803-DQA1^*∗*^0103-DQB1^*∗*^0601
Kang et al. [36]	1993	Japanese	82 (33/49)	DRB1^*∗*^0405-DRB4^*∗*^0101-DQA1^*∗*^0301-DQB1^*∗*^0401
Roitberg-Tambur et al. [43]	1993	Jews (Israel)	275 (17/258)	DQA1^*∗*^001-DQA1^*∗*^0201-DQB1^*∗*^0501
Roitberg-Tambur et al. [43]	1993	Greek	76 (22/54)	DQA1^*∗*^0501
Portales et al. [42]	1994	Spanish	286 (30/256)	HLA-Cw7 DR3 DR11
Wang et al. [32]	1997	Chinese	206 (70/136)	DR3, DR52, DR2, DR5, and DR9
Jean et al. [38]	1998	French	242 (42/200)	DRBI^*∗*^1501^*∗*^-0301-DQB1^*∗*^0201^*∗*^-0602
Rishmueller et al. [33]	1998	Australian	244 (80/164)	DR3-DQA1^*∗*^0501-DQB1^*∗*^02
Bolstad et al. [34]	2001	Norwegian Caucasian	95 (31/64)	DRB1^*∗*^03-DQB1^*∗*^02-DQA1^*∗*^0501
Nakken et al. [40]	2001	Norwegian Caucasian	210 (29/181)	DRB1^*∗*^0301
Anaya et al. [39]	2002	Colombian	149 (73/76)	DRB1^*∗*^0301
Gottenberg et al. [35]	2003	French	371 (149/222)	DRB1^*∗*^03
Manoussakis et al. [44]	2004	Greek	301 (55/246)	DRB1^*∗*^0301
Kovács et al. [37]	2006	Hungarian	98 (48/50)	DQB1^*∗*^0201-DRB1^*∗*^03-DQB1^*∗*^0501
Cruz-Tapias et al. [21]	2012	Meta-analysis	7636 (1166/6470)	DQA1^*∗*^0501-DQB1^*∗*^0201-DRB1^*∗*^0301-DQA1^*∗*^0201-DQA1^*∗*^0301-DQB1^*∗*^0501
Li et al. [46]	2013	Chinese	5622 (1845/3777)	HLA class II locus
Lessard et al. [45]	2013	Caucasian	10916 (4712/6204)	HLA class II locus

**Table 2:** Associations of non-HLA genetic locus with Sjögren's syndrome.

Gene/chromosome	Polymorphism	Population	Sample size (patients/controls)	*p* value	Relative risk	Study/year
Interferon pathways						
IRF5/Chr7	rs2004640	Caucasians	364 (210/154)	0.01	1.93	Miceli-Richard et al. 2007 [51]
	rs10488631 CGGGG promoter insertion/deletion	Norwegian/Swedish	1079 (368/711)	2.4 *∗* 10^−5^	1.49	Nordmark et al. 2009 [52]
	CGGGG promoter insertion/deletion	Caucasians	824 (385/439)	6 *∗* 10^−6^	2.00	Miceli-Richard et al. 2009 [53]
IRF5/TNPO3/Chr7	rs13246321	Norwegian/Swedish	1072 (540/532)	5.5 *∗* 10^−6^	1.70	Nordmark et al. 2011 [54]
	rs3757387	Caucasians	10916 (4712/6204)	2.73 *∗* 10^−19^	1.44	Lessard et al. 2013 [45]
STAT4/Chr2	rs7574865	Caucasians	1232 (120/1112)	0.01	1.47	Korman et al. 2008 [55]
	rs7582694	Norwegian/Swedish	1079 (368/711)	0.0014	1.41	Nordmark et al. 2009 [52]
	rs7574865	Colombian/German	800 (277/523)	7.7 *∗* 10^−6^	1.40	Palomino-Morales et al. 2010 [56]
	rs7582694	Norwegian/Swedish	1072 (540/532)	7 *∗* 10^−4^	1.40	Nordmark et al. 2011 [54]
	rs10168266	Chinese	5622 (1845/3777)	1.77 *∗* 10^−17^	1.44	Li et al. 2013 [46]
	rs10553577 rs13426947	Caucasians	10916 (4712/6204)	6.8 *∗* 10^−15^ 9.45 *∗* 10^−9^	1.43 1.32	Lessard et al. 2013 [45]
IL12A/Chr3	rs485497 rs583911	Caucasians	10916 (4712/6204)	1.17 *∗* 10^−10^ 9.88 *∗* 10^−9^	1.30 1.27	Lessard et al. 2013 [45]
NCR3/NKp30/Chr6	rs11575837	French/Scandinavian	1902 (1010/892)	0.0039	0.48	Rusakiewicz et al. 2013 [57]
	rs2736191			0.0019	0.56	
PTPN22/Chr1	rs2476601	French	3.55 (183/172)	ns	ns	Ittah et al. 2005 [58]
		Colombian	378 (70/308)	0.01	2.42	Gomez et al. 2005 [59]

B-cell function						
BLK-FAM167A/Chr8	rs12549796	Norwegian/Swedish	1072 (540/532)	4.7 *∗* 10^−4^	1.37	Nordmark et al. 2011 [54]
	rs7812879	Chinese	1152 (555/597)	0.045	—	Sun et al. 2013 [60]
	rs2736345 rs2729935 rs6998387	Caucasians	10916 (4712/6204)	4.97 *∗* 10^−10^ 6.85 *∗* 10^−10^ 7.96 *∗* 10^−8^	1.30 1.30 1.26	Lessard et al. 2013 [45]
CXCR5/Chr11	rs7119038 4936443	Caucasians	10916 (4712/6204)	1.0 *∗* 10^−8^ 6.82 *∗* 10^−8^	0.74 0.75	Lessard et al. 2013 [45]
BAFF/Chr13	−2841 T→C. −2704 T→C. −2701 T→A. −871 C→T	Caucasians	259 (123/136)	<0.001	—	Nossent et al. 2008 [61]
	rs1224141 rs12583006 rs9514828 rs1041569 rs9514827	Greek	330 (193/137)	<0.05	—	Nezos et al 2014 [62]
GTF2I/chr7	rs117026326	Chinese	5622 (1845/3777)	1.31 *∗* 10^−53^	2.20	Li et al. 2013 [46]
EBF1/Chr5	rs3843489	Norwegian/Swedish	1072 (540/532)	9.9 *∗* 10^−5^	1.68	Nordmark et al. 2011 [54]
Ox40L-TNFSF4/Chr1	rs1234315	Norwegian/Swedish		7.4 *∗* 10^−4^	1.34	Nordmark et al. 2011 [54]
	rs2205960 rs1234313	Chinese	643 (250/393)	<0.05	—	Kong et al. 2013 [63]

NF-*κ*B pathway						
TNFAIP3/chr6	rs2230926	Caucasians	(18/397)	0.038	3.38	Musone et al. 2011 [64]
	rs5029939	Chinese	5622 (1845/3777)	7.75 *∗* 10^−5^	1.67	Li et al. 2013 [46]
	rs6933404 rs35926684	Caucasians	10916 (4712/6204)	6.53 *∗* 10^−8^ 7.21 *∗* 10^−8^	1.26 1.26	Lessard et al. 2013 [45]
	rs2230926	Caucasians	1025 (574/451)	0.01	3.36	Nocturne et al. 2013 [65]
TNIP1/Chr5	rs6579837 rs7732451	Caucasians	10916 (4712/6204)	3.30 *∗* 10^−8^ 5.32 *∗* 10^−7^	1.43 1.34	Lessard et al. 2013 [45]
	rs3792783 rs7708392	Scandinavian/British	5565 (1105/4460)	3.4 *∗* 10^−5^ 1.3 *∗* 10^−3^	1.33 1.21	Nordmark et al. 2013 [66]
LTA/LTB/TNF gene clusters	rs1800629	Norwegian/Swedish	1060 (527/532)	1.6 *∗* 10^−11^	—	Bolstand et al. 2012 [67]
	rs909253			4.42 *∗* 10^−8^	—	
BAFF-R/Chr22	Ηis159Tyr	Greek	427 (247/180)	0.01	2.75	Papageorgiou et al. 2015 [68]
